# Extracellular translationally controlled tumor protein promotes colorectal cancer invasion and metastasis through Cdc42/JNK/ MMP9 signaling

**DOI:** 10.18632/oncotarget.10315

**Published:** 2016-06-28

**Authors:** Bin Xiao, Daxiang Chen, Shuhong Luo, Wenbo Hao, Fangyan Jing, Tiancai Liu, Suihai Wang, Yan Geng, Linhai Li, Weiwen Xu, Yajie Zhang, Xiaoqing Liao, Daming Zuo, Yingsong Wu, Ming Li, Qiang Ma

**Affiliations:** ^1^ State Key Laboratory of Organ Failure Research, Institute of Antibody Engineering, School of Biotechnology, Southern Medical University, Guangzhou, 510515, Guangdong, China; ^2^ RayBiotech, Inc., Guangzhou 510600, China; ^3^ RayBiotech, Inc., Norcross, GA 30092, USA; ^4^ Department of Anorectal Surgery, Nanfang Hospital, Southern Medical University, Guangzhou, Guangdong Province, 510515, China; ^5^ Department of Intensive Care Unit, 303 Hospital of People's Liberation Army, Nanning 530021, China; ^6^ Department of Laboratory Medicine, Guangzhou General Hospital of Guangzhou Military Command of PLA, Guangzhou, Guangdong 510010, China; ^7^ Division of Clinical Immunology Laboratory, Nanfang Hospital, Southern Medical University, Guangzhou, Guangdong Province, 510515, China; ^8^ Department of Immunology, School of Basic Medicine, Southern Medical University, Guangzhou, 510515, China

**Keywords:** extracellular TCTP, metastasis, Cdc42, p-JNK

## Abstract

The translationally controlled tumor protein (TCTP) can be secreted independently of the endoplasmic reticulum/Golgi pathway and has extrinsic activities when it is characterized as the histamine releasing factor (HRF). Despite its important role in allergies and inflammation, little is known about how extracellular TCTP affects cancer progression. In this study, we found that TCTP was overexpressed in the interstitial tissue of colorectal cancer (CRC) and its expression correlated with poor survival, high pathological grades and metastatic TNM stage in CRC patients. TCTP expression was greater in metastatic liver tissue than in primary tumors and was increased in highly invasive CRC cells. We demonstrated that the expression of TCTP was regulated by HIF-1α and its release was increased in response to low serum and hypoxic stress. Recombinant human TCTP (rhTCTP) promoted the migration and invasiveness of CRC cells *in vitro* and contributed to distant liver metastasis *in vivo*. Furthermore, rhTCTP activated Cdc42, phosphorylated JNK (p-JNK), increasing the translocation of p-JNK from the cytoplasm to the nucleus, as well as the secretion of MMP9. In addition, the expression of TCTP positively correlated with that of Cdc42 and p-JNK in clinical CRC samples. The silencing of Cdc42, JNK and MMP9 significantly inhibited the Matrigel invasion of rhTCTP-enhanced CRC cells. Collectively, these results identify a new role for extracellular TCTP as a promoter of CRC progression and liver metastases via Cdc42/JNK/MMP9 activation.

## INTRODUCTION

Colorectal cancer (CRC) is one of the most common malignancies with high rates of morbidity and mortality. More than half of CRC patients will suffer from systemic tumor metastases, most commonly liver metastases, which is the leading cause of death [1]. The clinical treatment of CRC remains challenging due to the lack of effective chemotherapy options for the treatment of late-stage metastatic disease. Therefore, the identification of specific markers for early diagnosis and therapeutic targets of CRC liver metastases is critical.

Translationally controlled tumor protein (TCTP), a highly conserved protein, is implicated in the tumorigenesis of various malignances [2]. In cancer, TCTP-centered program, including regulation of the tumor suppressor p53 and activation of components of the mTOR pathway, are involved in both subversion of the cancer stem cell compartment and the tumor reversion program [3]. TCTP also inhibits cell apoptosis by binding to Mcl-1 and blocking its ubiquitination [4, 5]. Our previous work showed that TCTP participated in CRC progression. Down-regulation of TCTP inhibited proliferation, migration and invasion activities of LoVo cells [6]. Lee et al. [7] also reported that TCTP can induce epithelial to mesenchymal transition and promotes cell migration, invasion and metastasis. However, data obtained in previous studies by Lee and our research group was limited to gene overexpression and knockdown strategies. Therefore, efforts to elucidate the direct role of TCTP in tumor cells and the underlying molecular mechanisms are imperative.

TCTP can be secreted and is well known for its well-established role as a histamine release factor (HRF) in triggering histamine release and cytokine production [2]. However, the correlation between extracellular TCTP and CRC metastasis has not been investigated. TCTP secretion is promoted by TSAP6 via a non-classical pathway [8]. In addition, TCTP increases the stability of hypoxia-inducible factor 1α (HIF-1α) by binding tumor suppressor VHL and promoting its degradation [9]. HIF-1 is a sequence-specific DNA-binding protein that acts as a master transcriptional activator of a broad range of genes involved in promoting adaptation and survival under hypoxic conditions [9]. Hypoxia is a hallmark of many human solid tumors and is associated with tumor progression [10]. The extent of hypoxia in a tumor may represent an independent indicator of poor prognosis [11]. Thus, we speculated that hypoxia-related stress might induce TCTP secretion to the outside of the cell and promote CRC invasion and metastasis.

In the present study, we have evaluated the role of extracellular TCTP on promoting tumor invasiveness of CRC cells and a new mechanism underlying the molecular behavior of extracellular TCTP-enhanced CRC cells. Our results suggest that TCTP could serve as a potential target for anti-metastatic therapeutic strategies in CRC.

## RESULTS

### TCTP is a potential indicator of CRC progression

**Figure 1:**
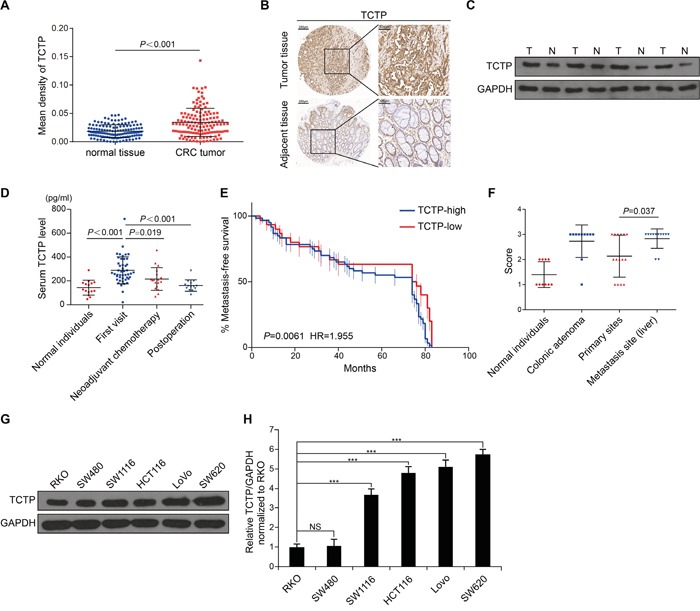
TCTP is a potential biomarker of CRC **A.** The expression levels of TCTP in 134 paired tumor tissues and adjacent tissues were detected by immunohistochemistry (IHC). **B.** Representative IHC images of TCTP expression in paired tumor and adjacent tissues. **C.** Western blot analysis of TCTP expression in four paired tumor and adjacent tissues. GAPDH served as a loading control. **D.** Bar graph showing TCTP expression in the serum of 93 CRC patients measured by ELISA. *P* value was calculated by One-Way ANOVA. **E.** Kaplan–Meier analysis of metastasis-free survival of 90 CRC patients, as a comparison of tumors expressing high TCTP versus low TCTP. *P*=0.0061 by long rank test. **F.** TCTP IHC staining score of normal individuals, colonic adenoma, primary sites, and metastasis sites. *P*=0.037 by Mann–Whitney U test. **G.** Western blot analysis of endogenous TCTP expression in six CRC cell lines. GAPDH served as a loading control. **H.** Relative TCTP mRNA levels (normalized to TCTP mRNA level in RKO) in six CRC cell lines. ***P<0.001 by One-Way ANOVA. NS: no significance.

TCTP expression was immunohistochemically determined in samples of paired CRC tumor and adjacent non-tumor tissues from 134 CRC patients. TCTP expression was significantly higher in CRC tumors than in adjacent non-tumor tissues (*P*<0.001) (Figure 1A and Figure 1B). The secretion of TCTP was increased in tumor tissues containing a higher level of intracellular TCTP (Figure 1B). Western blot analysis using total protein extracted from four paired tumor and adjacent non-tumor tissues of CRC patients showed similar results, with higher TCTP expression levels in tumors than in adjacent normal tissues (Figure 1C). Measurement of serum TCTP showed significantly higher levels in 93 first visit CRC patients than in individuals without CRC. (Figure 1D). In patients who underwent neoadjuvant chemotherapy or operative treatment, serum TCTP levels were significantly reduced compared with those of first visit CRC patients (Figure 1D). Analysis of TCTP expression according to the clinical parameters of CRC patients, such as age, gender, lymphoma metastasis, tumor size, pathological grade, American Joint Committee On Cancer (AJCC) stage, and Tumor Node Metastasis (TNM) stage showed that TCTP expression was positively associated with high pathological grades (*P*=0.014) and metastatic TNM stage (stage IV, *P*=0.006) (Table 1). Kaplan–Meier analysis revealed that high TCTP expression was significantly correlated with poor metastasis-free survival in 90 CRC patients (Figure 1E). TCTP expression at the site of liver metastasis was higher than that at the primary tumor site (Figure 1F), suggesting a role of TCTP in liver metastasis. TCTP expression at the protein and mRNA levels correlated with increased metastatic potential in the six CRC cell lines [12–14], which was consistent with our previous report [6] (Figure 1G and Figure 1H). Taken together, these results indicated that TCTP is a potential biomarker for CRC and plays a pivotal role in the progression and metastasis of CRC.

**Table 1:** Relationship between TCTP expression and clinicopathologic features of CRC patients

Features	Total number	High expression	Low expression	P	λ^2^
TCTP	134	86	48		
Age (years)				0.092	2.844
≤60	41	22	19		
>60	93	64	29		
Gender				0.739	0.111
Male	70	44	26		
Female	64	42	22		
Lymphoma metastasis				0.731	0.118
Positive	56	35	21		
Negative	78	51	27		
Tumor size (cm)				0.638	0.222
<5	51	34	17		
≥5	83	52	31		
Pathological grades				0.014	−2.458
I	15	5	10		
II	98	65	33		
III	21	16	5		
AJCC stage				0.834	−0.209
I-II	15	8	7		
	61	42	19		
III-IV	45	29	16		
	13	7	6		
TNM stage				0.006	7.491
I-III	113	67	46		
IV	21	19	2		

### The expression and secretion of TCTP are enhanced under low serum and hypoxic conditions

**Figure 2:**
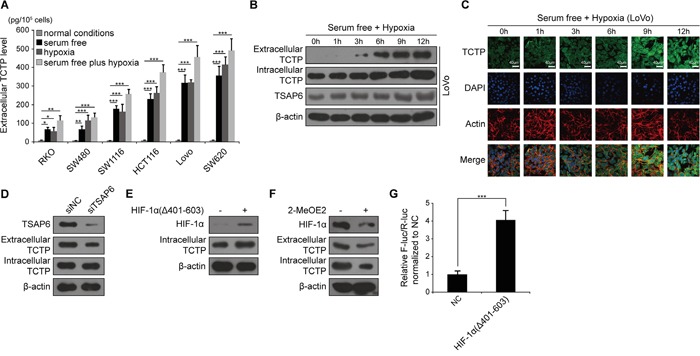
TCTP is secreted under low serum and hypoxic conditions **A.** Extracellular TCTP secreted from different medium-conditioned CRC cell lines was measured by ELISA. *P<0.05, **P<0.01, ***P<0.001 by One-Way ANOVA. **B.** Western blot analysis of the expression levels of extracellular TCTP, intracellular TCTP, and TSAP6 in LoVo cells under serum free plus hypoxic conditions. **C.** Expression of intracellular TCTP under serum free and hypoxic conditions at the indicated time points was observed under a confocal microscope. Representative photographs are shown. TCTP, FITC dye (green); F-actin, Rhodamine B dye (red); nuclear, DAPI dye (blue). Scale bar: 40 μm. **D.** Expression and secretion of TCTP mediated by silencing of TSAP6 under serum free plus hypoxic conditions. **E.** HIF-1α (Δ401-603) promotes TCTP expression under normal conditions. **F.** 2-MeOE2 inhibits TCTP expression and secretion under serum free plus hypoxic conditions. **G.** Relative luciferase activity was measured by transfection of either pENTER, pGL6-pTCTP-luc and pRLTK or pENTER-HIF-1α (Δ401-603), pGL6-pTCTP-luc and pRLTK for 24h under normal conditions. F-luc: Firefly luciferase. R-luc: Renilla luciferase. NC: negative control.

Traditionally, TCTP is released from a series of immune cells (basophils [15], eosinophils [16] and thymus [17]) and some types of cancer cells (prostate cancer [18]). To verify whether TCTP could be secreted from CRC cells, TCTP concentration was determined by ELISA in the culture supernatant of six CRC cell lines cultured under normal conditions (10%FBS and normoxia), in serum-free medium (1%FBS and normoxia), in hypoxic (10%FBS and 1%O_2_) conditions and in serum-free medium plus hypoxia (1%FBS and 1%O_2_). As shown in Figure 2A, the secretion of TCTP from hypoxia-conditioned cells was significantly higher than that from cells grown under normal conditions in the same cell line and was further enhanced in combination with serum deprivation (1%FBS). Under the same conditions, the secretion of TCTP was positively associated with the metastatic potential of the six CRC cells. Further western blot analysis confirmed the TCTP secretion pattern from LoVo and HCT116 under the above four culture conditions (Supplementary Figure S1A). We analyzed the secretion of TCTP in LoVo and HCT116 cells grown in the serum-free plus hypoxia conditions (Figure 2B and Supplementary Figure S1B). In both cell lines, the release of TCTP increased in a time-dependent manner in parallel with the up-regulation of intracellular TCTP and TSAP6, a TCTP interacting protein that facilitates the export of TCTP [8]. Using immunofluorescence to visualize intracellular TCTP, we observed a gradual increase in intracellular TCTP levels in LoVo and HCT116 cells (Figure 2C and Supplementary Figure S1C). When TSAP6 was silenced, the export of TCTP was inhibited under low serum plus hypoxic conditions. But the expression of intracellular TCTP did not change (Figure 2D). To further analyze the transcriptional regulation of TCTP under ischemic and hypoxic conditions, we evaluated the regulation of HIF-1α, a hypoxic biomarker, on TCTP expression. We generated an oxygen-dependent degradation domain (ODD)-deficient HIF-1α (HIF-1α-Δ401-603) expression vector which stably expresses and confers the DNA binding activity under normoxia which is equal to the activity of full length HIF-1 α under hypoxic conditions [19]. HIF-1α (Δ401-603) promotes TCTP expression under normal conditions 24h post transfection (Figure 2E). Using 2-methoxyestradiol (2-MeOE2) to inhibit HIF-1α expression under low serum and hypoxic conditions for 12h, TCTP expression and its subsequent export significantly decreased (Figure 2F). Dual luciferase reporter assays further showed that HIF-1α (Δ401-603) increased the luciferase activity driven by the TCTP promoter (promoter region: −200/0) by greater than 4 fold (Figure 2G). These results indicate that ischemia and hypoxia promote the expression and secretion of TCTP by activation of HIF-1α.

### Extracellular TCTP induces cell migration and invasion *in vitro*

**Figure 3:**
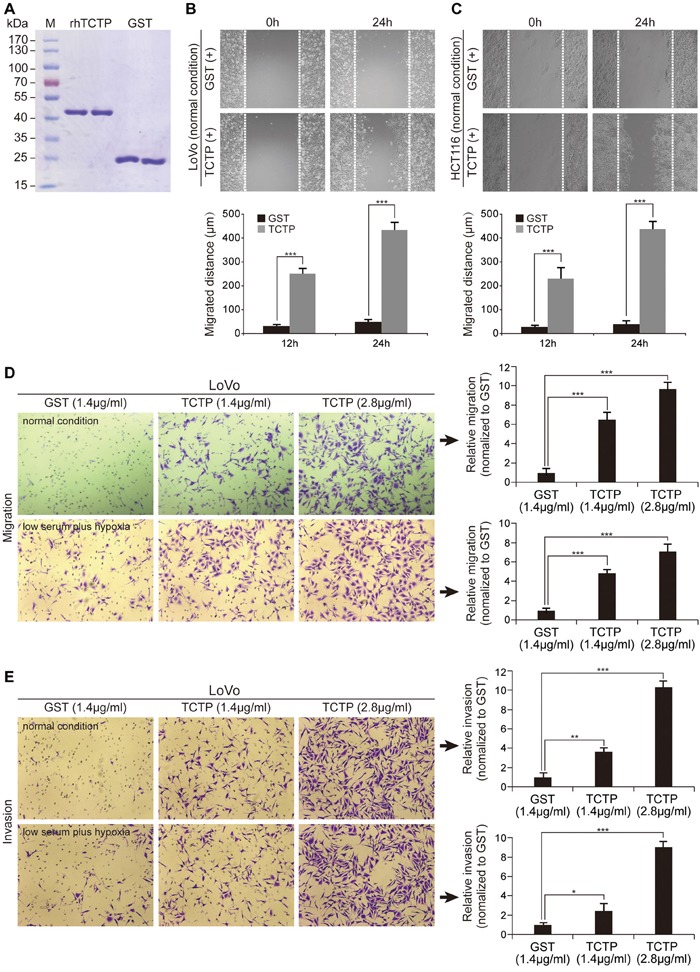
Extracellular TCTP promotes cell migration and invasion **A.** Coomassie blue staining of purified rhTCTP and prokaryotic GST. **B.** Wound closure of LoVo cells induced by rhTCTP or GST stimulation was measured from 0 to 24h. Migration distances are shown (right). ***P< 0.001. **C.** Cell migration of HCT116 cells induced by rhTCTP or GST stimulation was determined by the wound-healing assay. Migration distances are shown (right). ***P< 0.001. **D.** Migration ability of LoVo cells induced by different concentrations of rhTCTP or GST was determined by the Transwell assay (left) either under normal conditions or low serum plus hypoxic conditions. Relative migration ability was normalized to the control GST group (right). The data set shown is representative of three experiments. Magnification: 100×. Error bar indicates SD. ***P< 0.001. **E.** Different concentrations of rhTCTP or GST induced LoVo cell invasion in a Boyden chamber invasion assay either under normal conditions for 24h or low serum plus hypoxic conditions for 12h. Representative images from three independent repeated experiments are shown (left panel). Relative invasion ability was normalized to the control GST group (right). Data represents mean ± SD. *P< 0.05, **P< 0.01, ***P< 0.001.

A recent report revealed that overexpression of intracellular TCTP induces EMT and contributes to metastasis [7]. In the present study, high intracellular TCTP levels were associated with high levels of TCTP secretion (Figure 1H, Figure 2A, Figure 2B and Figure 2C) and we hypothesized that extracellular TCTP would share similar pro-metastatic functions. We generated a recombinant human TCTP (rhTCTP) fused to a glutathione S-transferase (GST) tag using a prokaryotic expression system. Meanwhile, GST alone was produced as a negative control (Figure 3A). In the wound healing assay, the scratched area of LoVo and HCT116 cells stimulated by rhTCTP for 24h closed faster than the control (Figure 3B and Figure 3C). In the Transwell migration assay, cells were grown under starvation conditions (RPMI1640+1%FBS) overnight and seeded in the top chamber. A significant migration of LoVo (Figure 3D) and HCT116 (Supplementary Figure S2A) cells towards rhTCTP in the bottom chamber was observed, compared with the control under normal conditions. Growing concentrations of rhTCTP resulted in increased numbers of migrated cells. Next, we examined the invasive capacity of LoVo and HCT116 cells that penetrated into the Matrigel in response to rhTCTP induction in a Transwell invasion assay. rhTCTP induced cell invasion in a dose-dependent manner (Figure 3E and Supplementary Figure S2B) under normal conditions. Importantly, enhanced migration and invasion abilities by rhTCTP were reproducible in the ischemic and hypoxic environment (Figure 3D, 3E and Supplementary Figure S2A, S2B). Taken together, these results demonstrated that extracellular TCTP could induce *in vitro* cell migration and invasion.

### Cdc42 and SAPK/JNK are activated by rhTCTP stimulation

**Figure 4:**
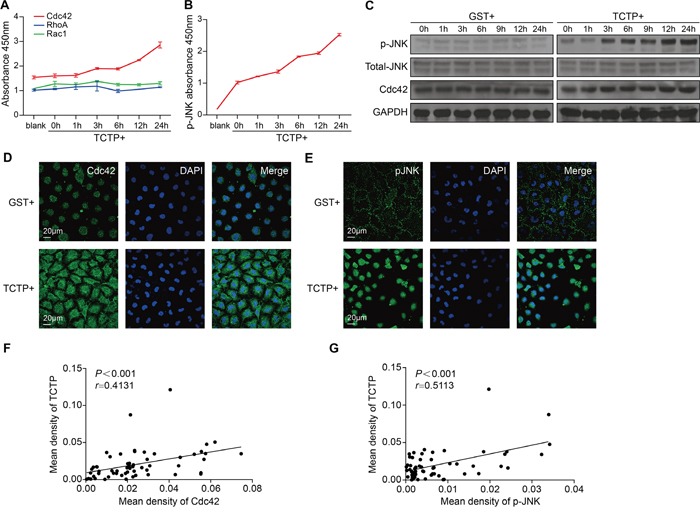
Cdc42 and JNK are activated by extracellular TCTP **A.** Small G-protein (Cdc42, Rac1, and RhoA) activation of LoVo cells by rhTCTP stimulation was measured at different time points by three independent G-LISA assays. LoVo cells were starved overnight and treated with rhTCTP (2.8μg/mL) for 0, 1, 3, 6, 12 and 24h. Lysates were assayed according to the instructions of the manufacturer. The degree of protein activation was determined by detecting absorbance at 490 nm. **B.** Activation of p-JNK in LoVo cells by incubation with rhTCTP at different time points was detected by the MAP kinase multi-target sandwich ELISA kit and signaling nodes multi-target sandwich ELISA kit. LoVo cells were starved overnight and treated with TCTP (2.8μg/mL) for 0, 1, 3, 6, 12, and 24h. Lysates were assayed according to the manufacturer's instructions. The degree of protein activation was determined by reading absorbance at 450 nm. **C.** Immunoblotting of p-JNK, total JNK, and Cdc42 in LoVo cells after incubation with GST or rhTCTP for the indicated time points. GAPDH level is as indicated. **D.** Immunofluorescence of Cdc42 induced by GST or rhTCTP stimulation for 24h. The cell nucleus (blue) was stained with DAPI. Scale bar: 20μm. **E.** Immunofluorescence of p-JNK induced by GST or rhTCTP stimulation for 24h. The cell nucleus (blue) was stained with DAPI. Scale bar: 20μm. **F.** Correlation between TCTP and Cdc42 expression levels in 60 human CRC samples. The Pearson correlation analysis was performed. **G.** Correlation between TCTP and p-JNK expression levels in 60 human CRC samples. *P*< 0.001 by Pearson correlation analysis.

To investigate the molecular mechanism underlying the pro-metastatic role of extracellular TCTP on CRC cells, we used G-LISA to screen three extensively characterized, small GTPase family proteins that play important roles in mediating cell metastasis [20–22]. Cdc42 was activated by rhTCTP and the RhoA and Rac1 levels were almost unchanged (Figure 4A). We also examined several convergence points and key regulatory proteins in signaling pathways using sandwich ELISA kits. The results showed that the expression of phospho-SAPK/JNK (Thr183/Tyr185) (p-JNK) was increased by rhTCTP stimulation in a time-dependent manner (Figure 4B), whereas other signals, including Akt1, phospho-Akt1 (Ser473), phospho-MEK1(Ser217/221), phospho-p38 MAPK (Thr180/Tyr182), phospho-Stat3(Tyr705), phospho-NFκB p65(Ser536), MEK1, SAPK/JNK, phospho-p44/42MAPK(Thr202/Tyr204) were not activated (data not shown). Immunoblot analysis confirmed that rhTCTP increased the level of p-JNK and Cdc42 with a strong activation of JNK at Thr183/Tyr185, but Akt and phospho-Akt1 (Ser473) were unchanged (Figure 4C and Supplementary Figure S3). Immunofluorescence assays showed that the Cdc42 fluorescent signal was elevated following rhTCTP stimulation, consistent with the immunoblotting results (Figure 4D). The translocation of p-JNK from the cytoplasm to the nucleus was activated by rhTCTP stimulation, as shown by immunofluorescent analysis (Figure 4E). The association of TCTP with Cdc42 and p-JNK was further examined by IHC. TCTP was positively correlated with Cdc42 expression in clinical CRC patient samples (Figure 4F). A significant positive correlation was also observed between TCTP and p-JNK expression in the same CRC patient samples (Figure 4G) and nuclear staining of p-JNK was observed in most cases (data not shown). These results indicate that Cdc42 and p-JNK were strongly activated by rhTCTP in conjunction with the translocation of p-JNK from the cytoplasm to the nucleus.

### Cdc42/JNK signaling mediates CRC cell migration and invasion induced by rhTCTP

**Figure 5:**
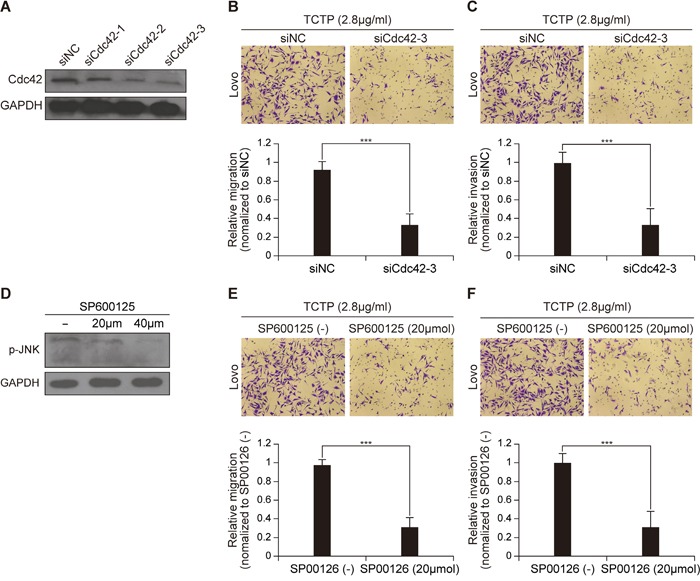
Cdc42 and JNK are responsible for extracellular TCTP induced cell migration and invasion **A.** Effect of three siRNAs targeting Cdc42 was detected by western blotting. **B.** siCdc42-3 inhibited rhTCTP-induced cell migration. The experiments were performed in triplicate. Error bar indicates SD. ***P< 0.001. **C.** TCTP-induced invasion ability of LoVo cells was reduced by siCdc42-3. The experiments were performed in triplicate. Error bar indicates SD. ***P< 0.001. **D.** Immunoblots measuring the expression of p-JNK in response to treatment with different concentrations of SP600125 in LoVo cells. **E.** SP600125 inhibited cell migration activated by rhTCTP. The experiments were performed in triplicate. Error bar indicates SD. ***P< 0.001. **F.** rhTCTP-induced invasion ability of LoVo was reduced by SP600125 treatment. The experiments were performed in triplicate. Error bar indicates SD. ***P< 0.001.

Cdc42 and JNK are closely associated with cancer cell metastasis [23–25]. To examine whether the activation of Cdc42 and JNK is responsible for extracellular TCTP-induced cell migration and invasion, the expressions of Cdc42 and p-JNK were knocked down using siRNA and the specific JNK inhibitor SP600125, respectively. Two siRNAs effectively downregulated endogenous Cdc42 expression (Figure 5A). The ability of rhTCTP-induced LoVo cells to migrate and invade by was significantly suppressed by siCdc42-3 by more than 2-fold (Figure 5B and Figure 5C). SP600125 treatment was used to reduce the phosphorylation of JNK in LoVo cells (Figure 5D). Increasing doses of SP600125 led to a decreasing number of migrated and invaded cells towards rhTCTP (Figure 5E and Figure 5F). Based on these results, we concluded that extracellular TCTP enhanced cell metastasis via the Cdc42/JNK pathway.

### JNK is phosphorylated by Cdc42 in the presence of rhTCTP and mediates the activation of MMP9

**Figure 6:**
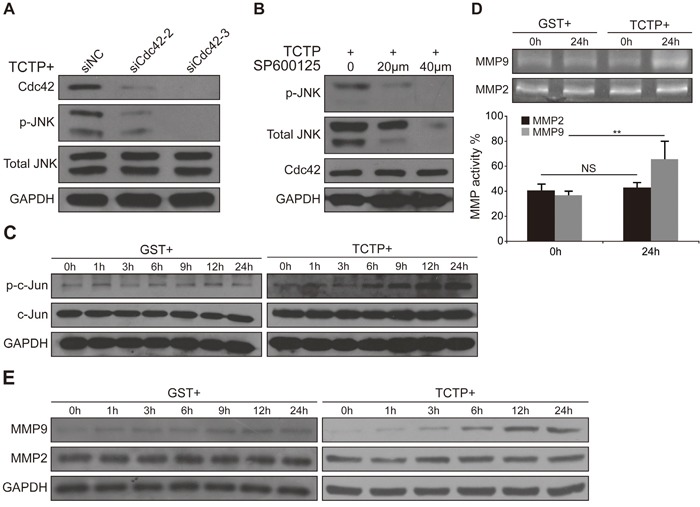
JNK was phosphorylated by Cdc42 and activated MMP9 expression **A.** Immunoblots showing that siCdc42 decreased the phosphorylation of JNK activated by rhTCTP. **B.** Immunoblotting shows that SP600125 had no effect on the expression of Cdc42. **C.** Western blot analysis of increasing c-Jun activation by rhTCTP stimulation in LoVo cells. **D.** Gelatin zymography assay showing elevated MMP9 activity in the culture medium of rhTCTP-stimulated LoVo cells. The MMP2 and MMP9 enzymatic activities were quantified. NS: no significance. **P< 0.01. **E.** Up-regulation of MMP9 expression in rhTCTP-stimulated LoVo cells, as evaluated by western blotting.

Cdc42 mediates the activation of JNK through MLK3 [26, 27]. Two MAP2K protein kinases, MKK4 and MKK7, directly phosphorylate JNKs on the threonine 183 (Thr183) and tyrosine (Tyr185) residues [28]. To examine whether the same signaling cascades were present in rhTCTP-stimulated CRC cells, Cdc42 was knocked down using siCdc42-3. The phosphorylation of JNK at Thr183/Tyr185 was significantly decreased in the presence of rhTCTP by siCdc42-3 (Figure 6A). Conversely, knockdown of p-JNK by SP600125 had no effect on the expression of Cdc42 (Figure 6B). We concluded that Cdc42 acted upstream of JNK and promoted the phosphorylation of JNK in the presence of rhTCTP stimulation.

When translocated to the nucleus, p-JNK binds to and activates the transcription factor AP-1, modulating the expression of a variety of substrates [27]. As shown in Figure 6C, the phosphorylation of c-Jun was increased in the presence of rhTCTP in a time-dependent manner compared with that of the control. Members of the MMP family are transcriptional targets of oncogenic AP-1 activation and are important for cancer invasion and metastasis [29]. We therefore investigated whether extracellular TCTP is required for the activation of MMPs in LoVo cells. The enzymatic activities of MMPs in the cell culture supernatant were measured using gelatin zymography assays. Compared with the GST group, a significant increase of MMP9 activity was found in the rhTCTP-stimulated supernatant of LoVo cells. However, MMP2 activity remained unchanged (Figure 6D). The enhanced activity was likely owing to increased MMP9 levels, as confirmed by a significant up-regulation of MMP9 protein expression in rhTCTP-stimulated LoVo cells (Figure 6E). The siRNA-mediated knockdown of MMP9 (Supplementary Figure S4A) showed that rhTCTP-induced migration and invasion of LoVo cells were also MMP9-dependent (Supplementary Figure S4B and S4C).

Taken together, these results indicate that Cdc42/JNK signaling is required for the activation of the AP-1/MMP9 axis in association with rhTCTP-induced cell metastasis.

### Extracellular TCTP promotes liver metastasis in mouse xenograft model

**Figure 7:**
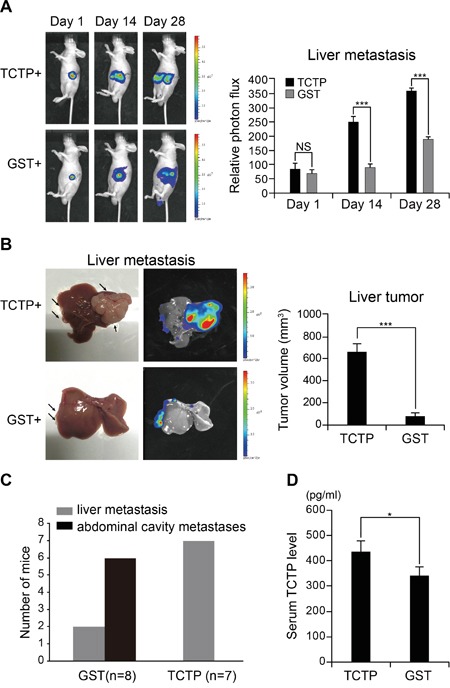
Extracellular TCTP induces liver metastasis in nude mice **A.** Balb/c nude mice were intraperitoneally transfused with rhTCTP or GST daily after the spleen was transfused with 1×10^6^ of LoVo-luc cells. The trace of the bioluminescent cells was monitored every 2 weeks. The proton flux was obtained by Xenogen IVIS LuminaXR software. Data represent the mean ± SD. **P< 0.001. NS: no significance. **B.** Balb/c nude mice were euthanized and photographed. Representative images of livers are shown (left). Tumor size was measured (right). Data represent the mean ± SD. **P< 0.001. **C.** Plot showing a statistical analysis of colonized organs between the GST and rhTCTP treatment groups. **D.** Serum TCTP level in GST or rhTCTP treated mice was measured by ELISA. *P*<0.05 by Student's t-test.

We investigated whether extracellular TCTP promotes tumor metastasis in nude mice. Initially, LoVo cells were engineered to express firefly luciferase by infection with lentivirus containing the corresponding recombinant plasmid. As liver metastasis accounts for the majority of deaths from CRC [30], these cells were injected into the spleens of nude mice (1×10^6^ cells per mouse) to establish a spleen-to-liver metastasis model. Mice received one daily intraperitoneal injection (3 mg/kg/mouse) of GST or rhTCTP proteins beginning on Day 1 for a period of four weeks. The fluorescent signal was monitored every 2 weeks. As shown in Figure 7A, tumor cells migrated faster to the liver in the rhTCTP-injected group than that in the control group. Unexpectedly, an increasing proton flux was observed in rhTCTP-treated mice, indicating a possible role of extracellular TCTP in cell proliferation (Figure 7A). No weight loss or other side effects were observed during rhTCTP treatment. When the livers were recovered from nude mice on day 28, we found that injection of rhTCTP led to a significant increase on tumor growth in the liver (Figure 7B). Statistically, all mice (7/7) showed liver metastases in response to rhTCTP treatment whereas control mice showed a lower incidence of liver metastases (2/8) and a higher incidence of abdominal cavity metastases (6/8) (Figure 7C). Taken together, our results indicate that extracellular TCTP promotes cell proliferation and liver metastasis *in vivo*, indicating a role of TCTP in organotropic metastases.

## DISCUSSION

**Figure 8:**
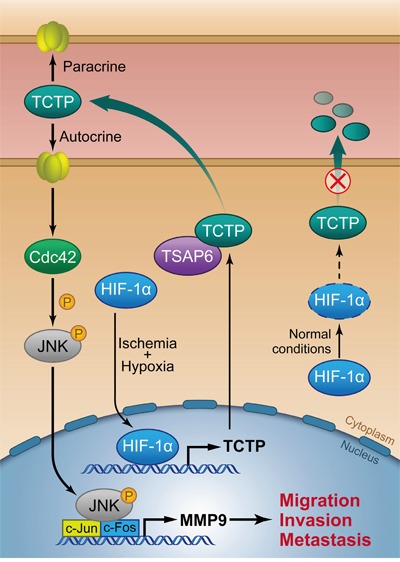
Schematic of ischemia and hypoxia-induced TCTP secretion and the role of extracellular TCTP in promoting CRC cell invasiveness and metastasis

Although mounting research focuses on extracellular functions of TCTP in asthma and allergies, the molecular mechanisms of extracellular TCTP responsible for cancer progression have not been reported. Here we provide evidence that ischemia and hypoxia-induced TCTP secretion promotes CRC cell invasion and liver metastases by activation of Cdc42/JNK/MMP9 signaling. In detail, we mapped a comprehensive overexpression of TCTP in tumor interstitial tissue, blood samples and metastasic liver tissue from CRC patients and in highly metastatic CRC cells (Figure 1). These results suggest that TCTP was secreted in CRC relevant tissues and entered the blood circulation system. Importantly, ischemia and hypoxia, two hallmarks of solid tumor, strongly induced TCTP secretion, indicating that the release of TCTP is the key step in CRC progression. Further support for this regulation is evidenced by the modulation of TCTP transcriptional activity by HIF-1α, a well-known hypoxic biomarker. Once secreted, TCTP may exert a direct autocrine and/or paracrine effect to promote the metastasis of CRC cells. Mechanistically, we found that Cdc42/JNK/MMP9 axis is activated and is imperative for the metastatic process. JNK was phosphorylated at Thr183/Tyr185 by Cdc42 in the presence of rhTCTP and, along with its nuclear translocation, promotes MMP9 activation, which initiates metastasis (Figure 8).

Combined detection of tumor-secreted proteins could significantly increase the sensitivity and specificity in detecting and monitoring tumorigenesis. Carcino-embryonic antigen (CEA) is the only serum marker which has been recommended for routine clinical detection of CRC. However, a recent study revealed that CEA is insufficiently sensitive to be used alone, even with a low threshold. It is therefore essential to augment CEA monitoring with another diagnostic modality in order to avoid missed cases [31]. In this study, we showed that TCTP level is higher in blood samples of first visit CRC patients (*P*<0.001), but decreased post-operatively (*P*<0.001) and following chemotherapy treatment (*P*=0.019). Similar to the close correlation of CEA level with TNM stage, pathological grades and liver metastasis [32–34], the same trend was also observed in high TCTP level towards these features in this study and in epithelial ovarian cancer. These results suggest that TCTP may serve as a predictor of CRC progression. Further research will be required to enlarge the clinical samples and try the combined usage of TCTP and CEA in detecting CRC.

Ischemic and hypoxic conditions are commonly found inside solid tumors. As we evaluated in this study, these conditions promote TCTP expression and secretion. We demonstrated transcriptional control of TCTP by HIF-1α. Under hypoxic conditions, the activity of PHDs and FIH1 was inhibited and HIF-1α subsequently binds to CREB-binding protein and the p300 complex (CBP/p300) to promote gene transcription [35]. It has been reported that the expression of TCTP is directly regulated by cAMP response element binding protein (CREB) transcription factors under normoxia [36]. The interactions of CBP with CREB and HIF-1α were detected by coimmunoprecipitation and the recruitment of CBP/CREB and HIF-1α/CBP complexes to two enhancer regions of neuroblast GLUT3 was required for GLUT3 transcription [37]. Thus, it is natural to assume the cooperation of CREB and HIF-1α, which are co-activated under hypoxia stress and promote TCTP expression.

TCTP is a component of the exosome, where it is packaged during secretion [8]. The exosome is one of the most abundant stromal components associated with metastasis [38]. Tumor-derived exosomes can train specific host tissues towards a pre-metastatic tendency by providing autocrine, paracrine, endocrine, and other signals [39]. They are taken up by organ-specific cells and remodel the pre-metastatic niche, guiding organotropic metastasis determined by distinct exosome integrin expression patterns [40]. Our *in vivo* metastasis assay revealed that extracellular TCTP can guide CRC cells colonizing the spleen to metastasize to the liver. This finding indicates that TCTP may also possess organotropic properties. However, the mechanism underlying the effect of TCTP on guiding liver metastases remains to be identified.

Cdc42 is a small GTPase of the Rho-subfamily which plays pivotal roles in cell morphology, migration, endocytosis, and cell-cycle progression. The rearrangement of the cytoskeleton induced by Cdc42 is important for metastasis [41]. TCTP is also involved in cell morphology regulation through its interaction with the actin cytoskeleton, revealing a feasible collaboration of TCTP and Cdc42. As a downstream factor of Cdc42, JNK is indirectly phosphorylated by Cdc42 and regulates AP-1 transcriptional activity by binding directly to the AP-1 promoter. Aberrant expression and activation of JNK are often observed in many cancer cell lines and in patient tissues [27]. The role of JNK is controversial, as it has been shown to be a positive regulator of CRC metastasis as shown in this study and other reports [14, 42], whereas other studies demonstrated a role of JNK as a tumor suppressor in CRC [43]. Studies in mice have demonstrated that the contribution of JNK is cell type and isoform specific, which may explain the seemingly opposite role of JNK in promoting cell survival and proliferation on one hand and cell death on the other [25]. Clinical assessments have demonstrated that MMP9 expression is associated with overall survival of patients with CRC liver metastases [44]. The overexpression of MMP9 can be stimulated by either extracellular or intracellular TCTP [7]. However, intracellular TCTP overexpression enhanced cell migration via activation of mTORC2/Akt/GSK3b/β-catenin, while extracellular TCTP had no effect on Akt and phospho-Akt1 (Ser473). Explanations for this discrepancy include the possibility that TCTP functions differently within cells and in the extrinsic space, or due to the fact that different cancer types were addressed in the two studies.

In conclusion, our findings suggest that extracellular TCTP could be considered as a novel biomarker for the clinical diagnosis of CRC. In consideration of the pro-metastatic and organotropic role of extracellular TCTP, developing novel TCTP inhibitors or antibodies capable of blocking either the transcription and translation or the secretion of TCTP is urgent. Our understanding of the downstream signaling targets of extracellular TCTP also offers opportunities for the design of anti-Cdc42/JNK/MMP9 therapeutic strategies.

## MATERIALS AND METHODS

### Cell culture and chemicals

CRC cell lines were purchased from the cell bank of the Chinese Academy of Science. Cells were cultured in RPMI 1640 (Cat: C11875500BT, Gibco) supplemented with 10% fetal bovine serum (FBS, Cat: 087-150, Wilsent) and 1% penicillin/streptomycin (Cat: V900929, Sigma Aldrich) at 37°C in an atmosphere of 5%CO_2_. Serum free conditions were established when cells were cultured in Minimal Essential Medium (MEM, Cat: 12571071). Hypoxic conditions (1%O_2_) were set up in a hypoxia incubator (Forma Scientific, Marietta, OH, USA) where N_2_ was applied to supplement the decreasing O_2_ level. A TPT1 Elisa kit (Cat: CSB-EL024134HU) was purchased from Cusabio (Wuhan, China). 2-Methoxyestradiol (Cat: S1233) was from Selleck.cn (Huston, Texas, USA). Phospho-SAPK/JNK (Thr183/Tyr185) Rabbit mAb (Cat: 4668P), Cdc42 Antibody (Cat: 2462), Akt antibody (Cat:9272), phospho-Akt (Ser473) antibody (Cat: 4060), c-Jun Mouse mAb (Cat: 2315), phospho-c-jun (Ser63) antibody (Cat: 2361), MMP-2 Rabbit mAb (Cat: 4022), MMP-9 Rabbit mAb (Cat: 13667) were purchased from Cell Signaling Technology (Boston, MA, USA). JNK mouse mAb (Cat: GB13018) was purchased from Goodbio Technology (Wuhan, China). HIF-1 antibody (BS3514), TSAP6 polyclonal antibody (Cat: BS6032), GAPDH polyclonal antibody (Cat: AP0063), β-actin polyclonal antibody (Cat: AP0060), Goat anti-Mouse IgG (H+L) –HRP (Cat: BS12478), and Goat anti-Rabbit IgG (H+L) –HRP (Cat: BS13278) were acquired from Bioworld Technology (St. Louis Park, MN, USA). Dulbecco's Modified Eagle Medium (DMEM, Cat: 11965118) was from Life Technologies (Grand Island, NY, USA). DNA Polymerase (Cat: R045A), T4 DNA ligase (Cat: 2011A), restriction enzymes (*Eco*R I, Cat: 1040S; *No*t I, Cat: 1166S) and competent cells (Cat: 9057) were purchased from Takara (Dalian, China). SP600125 (Cat: S5567) was purchased from Sigma-Aldrich (St. Louis, MO, USA). Small interfering RNAs (siRNA) were synthesized by Genepharma (Suzhou, China). All other reagents were analytical grade.

### Western blotting

Stimulated or transfected cells were lysed in RIPA buffer. The lysates were sonicated and pelleted by centrifugation. Protein concentration was measured using the BCA protein assay kit (Thermo Fisher Scientific, Waltham, MA, USA). The lysates were collected with 5×loading buffer, boiled for 15 min, and loaded equally on SDS-10% polyacrylamide gels. Following electrophoresis, the proteins were transferred to PVDF membranes at a constant 100V for 90 min. The PVDF membranes were blocked with 5% nonfat milk for 1h and probed with respective primary antibodies at 4°C overnight. After three washes with PBST for 15 min each, the PVDF membranes were incubated with an HRP-conjugated anti-rabbit (mouse) antibody for 1h. Finally, the membranes were washed with PBST three times for 15 min each. The blots were visualized by ECL chemiluminescence reagent using FUJI SUPER RX film. Each western blot was repeated in triplicate.

### Immunofluorescence

Cells were passaged until reaching 80% confluence and then treated with rhTCTP under hypoxic and serum-free conditions for 0h, 1h, 3h, 6h, 9h and 12h respectively. Cells were then fixed with 4% paraformaldehyde for 15 min, permeabilized with 0.25% Triton X-100 for 10 min at room temperature, and blocked with PBST for 30 min. After washing with PBS, the cells were incubated with an anti-TCTP antibody (1:10000, Cat: 8441. Cell Signaling Technology, Boston, USA), and probed with anti-rabbit IgG Alexa Fluor 488 (Cat: SA5-10078. Life Technologies, Grand Island, USA) for 1h at ambient temperature. The cells were washed in PBS and treated with DAPI for 10 min. For phalloidin staining, cells were washed in PBS and incubated with phalloidin (1:100, Cat: PHDG1. Cytoskeleton, Denver, USA) for 20 min at room temperature. The fluorescent signals of both assays were observed using a Leica inverted fluorescence microscope (Cat. TCS SP5. Leica, Germany).

### Real-time quantitative PCR (qPCR)

Total RNA from LoVo cells was extracted using the Trizol method [45]. cDNA was synthesized using the cDNA Synthesis Supermix for qPCR (Cat. AT341-01. Transgen, Beijing, China) following the manufacturer's instructions. PCR amplification was performed in a reaction volume of 20μL containing Sybr green on an Applied Biosystems 7500 under the following conditions: 94°C for 30s, followed by 42 cycles of 94°C for 5s and 60°C for 34s. The final results were calculated using the 2-ΔΔCt method to analyze differences in mRNA expression levels between specimens. Primers used to amplify TCTP in this experiment were as follows:

Forward primer: AGGGGCTGCAGAACAAATCA

Reverse primer: AGACAGAAAGCGCAGGGATT

### Dual luciferase reporter assay

The fragment of the TCTP promoter (promoter sequence: −200/0) was cloned into pGL6-luc (Firefly luciferase) to generate pGL6-pTCTP-luc. LoVo cells were cultured in 24-well plates (1 × 10^5^ cells/well) and co-transfected with 300 ng pGL6-pTCTP-luc, 40 ng pRLTK (Renilla-luciferase) and either 1μg pENTER or pENTER-HIF-1α (Δ401-603). 24 h post transfection, the cells were lysed and luciferase activity was measured with the Dual-Luciferase Reporter Assay System (Cat. E1910. Madison, WI, USA).

### Preparation of recombinant human TCTP and GST

The cDNA from LoVo cells was used as template to amplify TCTP by reverse-transcription PCR. PCR products were cloned into pGEX4T-1 and transferred into *E. coli* BL21 by incubation at 42°C for 1 min. A single clone was selected to be induced by isopropyl-β-d-thiogalactoside. The soluble forms of TCTP and GST were obtained by sonication extraction and purified using GSH beads (Cat. 70601-5. Beaver Nano Technology, Suzhou, China) and dialyzed against PBS. Purified TCTP and GST were sterilized using a 0.22-μm millipore membrane for experimental use.

### Cell migration and invasion assays

The effects of GST, rhTCTP, siRNA, and JNK inhibitor on cell migration were measured using a 6.5-mm Transwell plate (Cat. 3422. Corning, NY, USA) with a 0.4-μm pore polyester membrane insert in a 24-well plate. Briefly, cells were grown under starvation conditions (RPMI1640+1%FBS) overnight. After trypsinization, cells were resuspended at a density of 1.5×10^6^/mL in DMEM supplemented with 1% FBS and a volume of 200μL was transferred to the upper chamber of the Transwell plate. The lower chamber was filled with DMEM containing 1% FBS and a gradient concentration of rhTCTP or GST. After 14h of incubation, cells were immobilized with 100% methanol for 10 min and stained with 0.1% crystal violet for 15 min. The non-migrating cells were removed with a cotton swab. For cell invasion assays, the experimental procedures were similar to those of the cell migration assay with two notable modifications. First, an invasion chamber 24-well plate (Cat. 354480. BD Biosciences, Franklin Lakes, USA) was used instead of the 6.5-mm Transwell plate. Secondly, the incubation time was prolonged to 20h for LoVo cells and to 24h for HCT1116 cells. For both assays, migrated cells were counted in six randomly selected fields under a microscope at a magnification of 400×. All assays were performed in triplicate.

### Wound-healing assay

The role of rhTCTP in the migratory ability of LoVo and HCT116 cells was determined using a wound-healing assay. Cells were seeded on 6-well plates and a wound was scratched in the cell lawn with a 200-μL pipette tip. The cells were washed with PBS three times to remove debris. Purified rhTCTP was added at a final concentration of 2.8μg/mL and images of wound closure were captured every 4 h for 24 h. All experiments were performed in triplicate.

### Small G-proteins and MAPK multi-target sandwich ELISA

Briefly, Small G-protein (RhoA, Cdc42 and Rac1) activation was measured using the Small G-proteins Activation Kits (Cat. BK124, Cat. BK127 and Cat. BK128. Cytoskeleton, Denver, USA). according to the manufacturer's instructions. The activation of MAPK target proteins was detected using a MAPK multi-target sandwich ELISA kit (Cat. 7272 and Cat. 7274. Cell Signaling Technology, Boston, USA) according to the manufacturer's instructions.

### siRNA synthesis and transfection

siRNA fragments were synthesized by Genepharma (Shanghai, China). The siRNA sequences are shown in Table 2. siRNA (40μmol) was transfected using Lipofectamine 2000 (Cat: 11668500. Life Technologies, Grand Island, USA) according to the manufacturer's instructions. Twenty-four hours later, cells were collected for western blotting, cell migration, or cell invasion assays as indicated in the respective sections.

**Table 2:** siRNA sequences used in this study

Target gene	siRNA sequences
TSAP6	siTSAP6: 5′-CUACUCUUCACUGUGCAGUTT-3′
	siNC: 5′-UUCUCCGAACGUGUCACGUTT-3′
Cdc42	siCdc42-1: 5′-CCGCUGAGUUAUCCACAAATT-3′
	siCdc42-2: 5′-CCUCUACUAUUGAGAAACUTT-3′
	siCdc42-3: 5′-GUGGAGUGUUCUGCACUUATT-3′
	siNC: 5′- UUCUCCGAACGUGUCACGUTT −3′
MMP9	siMMP9: 5′-AGUACUGGCGAUUCUCUGAGGUTT-3′
	siNC: 5′-CGUUGGCGACGUUAUAAGCUUGTT-3′

### *In vivo* metastasis assay

Male balb/c nude mice, aged from 6 to 8 weeks, were purchased from the Laboratory Animal Center of the Southern Medical University. To study the effect of TCTP on liver metastasis in CRC cells, LoVo cells were infected with a lentivirus luciferase vector generating LoVo-luciferase (LoVo-luc). LoVo-luc cells (1×10^6^) were injected into the spleens of nude mice by surgical operation. Purified rhTCTP was transfused by daily intraperitoneal injection, starting on the day after surgical operation, at a dose of 3 mg/kg and the trace of fluorescent cells was monitored every 2 weeks using an IVIS Lumina II (Caliper Life Sciences, USA). On day 28, the mice were sacrifced and their livers were surgically excised, photographed, and measured. All animal procedures were conducted in compliance with ethical standards and approved by the Animal Care Committee of Southern Medical University.

### CRC patient samples, immunohistochemistry, and scoring

Human CRC tissue microarray slides (Cat: HCol-Ade180Sur-08, HCol-Ade080CD-01 and HCol-Ade060CS-01) were supplied by Shanghai OUTDO Biotech (Shanghai, China). A total of 268 CRC samples with adjacent normal colon tissues were obtained with detailed patient information, including age, gender, metastasis status, pathological grades, tumor size, TNM stage, and AJCC stage. Of these, 90 cases had undergone surgical operation between June 2007and April 2008 with follow-up examinations between June 2007 and September 2014. The mean follow-up period was 57 months, ranging from 2 to 87 months. The human tissue samples were used on the bases of the guidelines of Nanfang Hospital. Written informed consents were signed by each participant prior to their inclusion in this study.

For immunohistochemical assays, tissue samples were dissected, fixed in 4% paraformaldehyde, and sliced into 4-mm paraffin-embedded pieces. After dewaxing, nonspecific peroxidase activity was blocked with 3% H_2_O_2_ for 15 min, followed by washing three times with PBS for 5 min per wash. Sections were then incubated in 5% BSA-PBS for 30 min, followed by probing with the respective primary antibodies (1:200 dilution for TCTP staining, 1:100 dilution for Cdc42, and 1:100 dilution for p-JNK) at 4°C overnight. Immunostaining continued with HRP-conjugated secondary antibodies for 4h at room temperature. Hematoxylin was then applied for nuclear counterstaining.

Sections were analyzed using Image Pro-Plus 6.0 Software. The expression of TCTP was scored according to the mean density (the ratio of the integral optical density to the total area). Positive expression was determined by obvious light-yellow or brownish yellow staining in the cytoplasm. The mean percentage of positive cells was classified into four categories: 0 for negative, 1 for <10%, 2 for 10–60%, and 3 for >60%. High and low protein expression was defined using the mean score of all samples as a cutoff point [46]. The correlation of TCTP expression with Cdc42 and p-JNK expression was analyzed by the Mann–Whitney U-test. Kaplan–Meier analysis and the long rank test were used to determine metastasis-free survival. The relationship between TCTP expression and clinical parameters of CRC patients was evaluated by the Pearson Chi-Square test.

### Gelatin zymography

Gelatin zymography was performed as previously described [47]. Briefly, cells were cultured for 24h in serum-free medium in the presence of either rhTCTP or GST. The culture medium was concentrated using centrifugal filters and separated on non-denaturing SDS/PAGE gels containing 0.1% gelatin. After running, the gel was washed in 1× Zymogram Renaturing Buffer (25 ml Triton X-100 in 1L deionized water) at room temperature for 30 min and then incubated in 1× Zymogram Developing Buffer (50 mM Tris-HCl, 5 mM CaCl2 2H2O, 0.02% Brij-35, 0.2 M NaCl, pH7.6) overnight. The gel was stained with Coomassie Blue R-250 for 30 min and destained with an appropriate Coomassie R-250 destaining solution (Methanol: Acetic acid: Water (50: 10: 40). The bright bands, which represent the MMPs activities, will appear against a dark background.

### Repeatability of experiments and statistical analysis

Each experiment was repeated at least three times. Data are presented as the mean ± SD unless otherwise stated. The Student's t-test was used to compare two groups of independent samples. A *P*-value<0.05 was considered statistically significant.

## SUPPLEMENTARY MATERIALS FIGURES


