# Supplementary material for: Extracellular translationally controlled tumor protein promotes colorectal cancer invasion and metastasis through Cdc42/JNK/ MMP9 signaling
Source: Oncotarget. 2016 Jun 28;7(31):50057–73. doi: 10.18632/oncotarget.10315 (PMC5226568; doi:10.18632/oncotarget.10315)
Supplement: Supplementary file 1 [file oncotarget-07-50057-s001.pdf]

Extracellular translationally controlled tumor protein promotes colorectal cancer invasion and metastasis through Cdc42/JNK/MMP9 signaling

SUPPLEMENTARY FIGURES

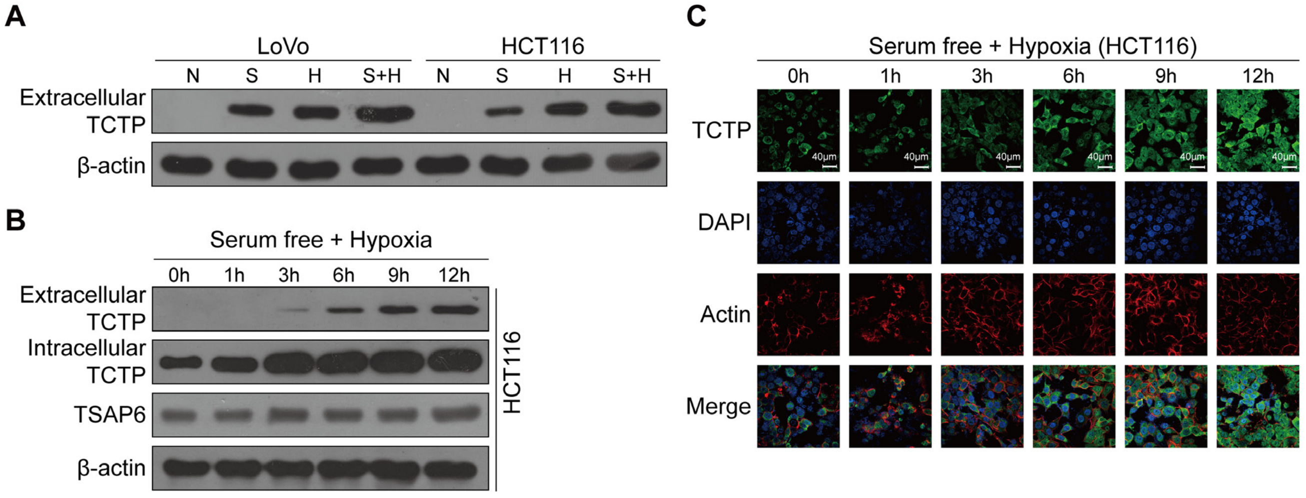

Supplementary Figure S1: A. Western blot analysis of extracellular TCTP secreted from LoVo and HCT116 cells from different culture conditions. N: normal conditions. S: serum free conditions. H: hypoxic conditions. **B.** Indicated gene expression and TCTP secretion in HCT116 cells at different time points under low serum plus hypoxic conditions. **C.** Immunofluorescence showing hypoxia-induced intracellular TCTP expression in HCT116 cells. TCTP, FITC dye (green); F-actin, Rhodamine B dye (red); nucleus, DAPI dye (blue). Scale bar: 40 µm.

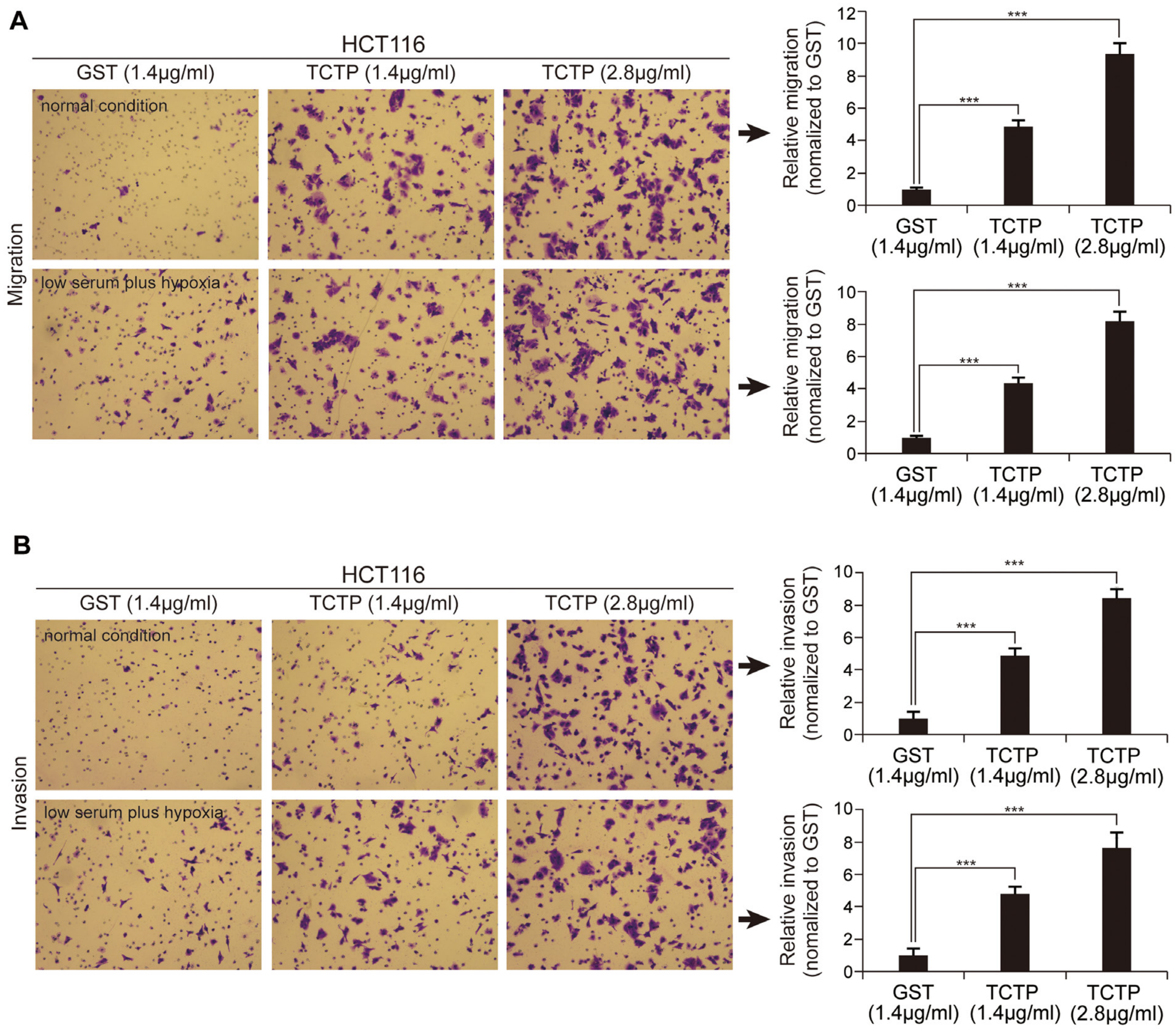

Supplementary Figure S2: A. Transwell migration assay of HCT116 cells stimulated by different concentrations of rhTCTP or GST either under normal conditions for 24h or low serum plus hypoxic conditions for 12h (left). Relative migration ability was normalized to the control GST group (right). Magnification: 100×. Error bar indicates SD. ****P*< 0.001. **B.** Invasive properties of HCT116 induced by different concentrations of rhTCTP or GST were evaluated by the Transwell invasion assay either under normal conditions for 24h or low serum plus hypoxia conditions for 12h. Representative images from three independent repeated experiments are shown (left panel). Relative invasion ability was normalized to the control GST group (right). Data represents the mean ± SD. ****P*< 0.001.

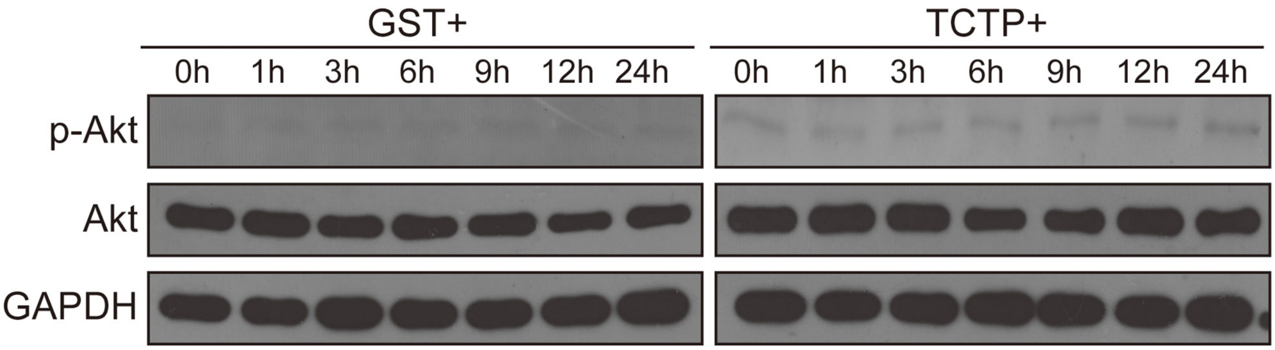

Supplementary Figure S3: Western blot analysis of the expression of Akt and phospho-Akt (Ser473) in rhTCTP-stimulated LoVo cells.

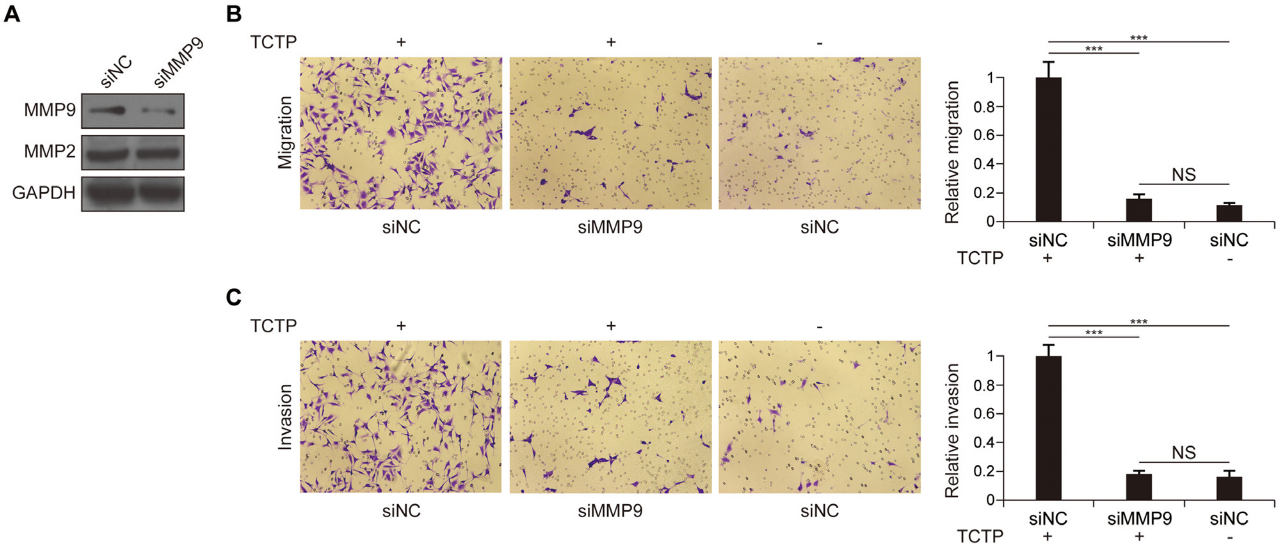

Supplementary Figure S4: A. Effect of siRNA targeting MMP9 was detected by western blotting. **B.** rhTCTP-induced migration ability of LoVo cells was inhibited by silencing MMP9. Magnification: 100×. Error bar indicates SD. ***P< 0.001. NS: no significance. **C.** Matrigel invasion of LoVo cells stimulated by rhTCTP decreased upon knockdown of MMP9. Magnification: 100×. Error bar indicates SD. ***P< 0.001. NS: no significance.
